# HEAD-MIP–(HEAlth Dialogues for patients with Mental Illness in Primary care)—a feasibility study

**DOI:** 10.1186/s40814-023-01391-2

**Published:** 2023-09-28

**Authors:** Veronica Milos Nymberg, Miriam Pikkemaat, Susanna Calling, Peter Nymberg

**Affiliations:** 1 Center for Primary Health Care Research, Department of Clinical Sciences, Lund University, Malmö, Sweden; 2 School of Health and Welfare, Halmstad University, Halmstad, Sweden

**Keywords:** Health Dialogue, Lifestyle, Mental illness, Primary care

## Abstract

**Background:**

Patients with mental illness have an increased risk of cardiovascular morbidity and mortality compared to the rest of the population, which is partly related to unhealthy lifestyle habits. To individualise lifestyle counselling in primary care, the Swedish-developed Health Dialogue (HD) can be used as an educative tool at recurrent measurement points with the goal to improve non-healthy lifestyle habits. HD has not been aimed specifically at patients with mental illness, and the effect of a systematic approach with repeated HDs in patients with mental illness in primary care has not been previously studied. The aim of this pilot study was to assess the feasibility of the study design for a larger-scale cohort study using repeated HDs focused on the improvement of lifestyle habits in patients seeking primary care due to anxiety, depression, sleeping problems or stress-related symptoms.

**Methods:**

Patients were recruited after a visit to a Primary Health Care Center due to mental illness between October 2019 until November 2021 and received a Health Dialogue, including an assessment of cardiovascular risk factors through a Health Curve. Specific feasibility objectives measured were dropout rate, time to follow-up, and risk improvement rate for different lifestyle changes.

**Results:**

A total of 64 patients were recruited and 29 (45%) attended a second HD, with a mean follow-up time of 15 months. All participants had at least one elevated cardiovascular risk level on the Health Curve for the assessed lifestyles. Risk level improvement rate was good except for tobacco use.

**Conclusion:**

Despite a higher dropout rate than expected, we suggest that the proposed methodology for a full cohort study within general practice of patients with mental illness in primary care is both acceptable to practice and feasible.

**Trial registration:**

NCT05181254. Registered January 6th, 2022. Retrospectively registered.

## Key messages regarding feasibility

- The Swedish-developed Health Dialogue has not previously been studied for the improvement of lifestyle habits in patients seeking primary care due to mental illness.
- Our results show higher drop-out rate than expected between baseline and follow-up, but acceptable proportion of follow-up attendees adopting recommended changes in lifestyle habits
- We suggest that the proposed methodology for a full cohort study within the general practice of patients with mental illness in primary care is both acceptable to practices and feasible.

## Background

**Fig. 1:**
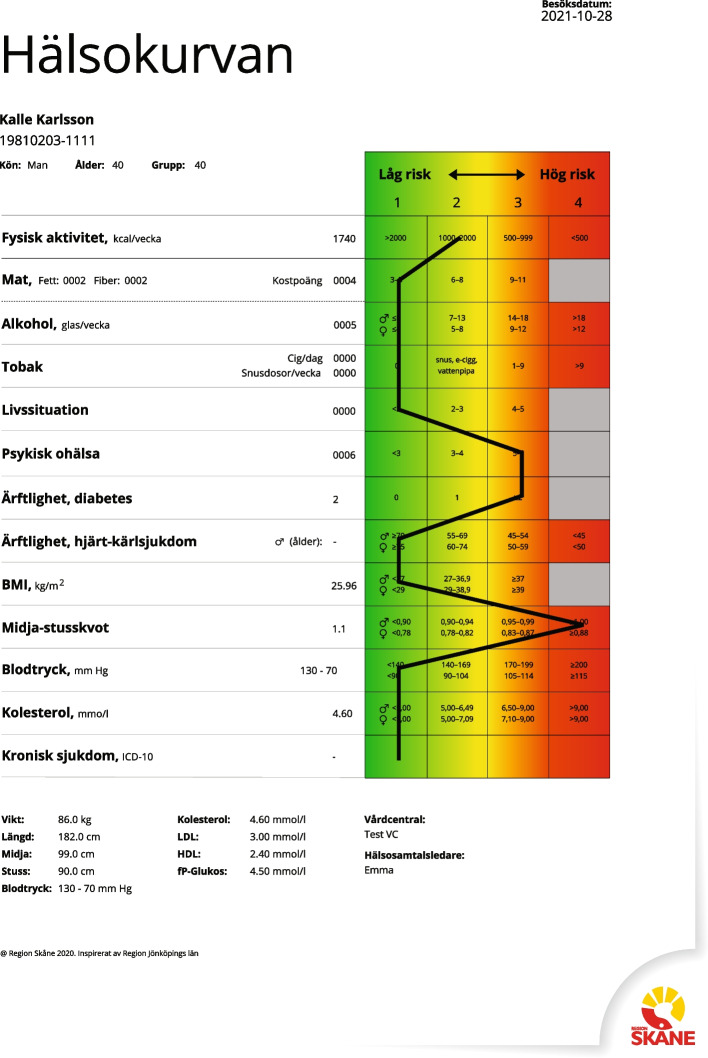
The Health Curve, with risk level assessment for different lifestyle areas on the summary page (original Swedish version)

Mental illness has negative effects on the individual’s well-being, as well as economic and social consequences [1], and might cause a range of socioeconomically patterned physical illnesses [2]. Patients with mental illness have an increased risk of cardiovascular events compared to the rest of the population, partly due to a higher prevalence of high blood pressure, diabetes, hypercholesterolaemia, and obesity [2–5]. Early detection of cardiovascular risk factors might therefore reduce the risk of metabolic and cardiovascular complications [6, 7]. While patients with mental illness are less prone to be compliant with preventive recommendations [8], they want to be encouraged by their healthcare provider to change their habits into a healthier lifestyle [9] in a similar way as the general population [10]. There is evidence for the effect of lifestyle interventions on improved mental health outcomes such as stress, anxiety, and depression for this group of patients [11–13]. However, the effect on cardiovascular risk factors is more controversial. A recent randomised controlled Swedish study showed that a single visit to a health coach did not improve exercise levels in 50-year-old individuals, and participants even rated their health as worse after the intervention compared with the control group [14]. However, there are indications that individualised programs provide better health outcomes compared with lifestyle screening in the general population [15]. Most studies with lifestyle coaching have been performed using multiple contacts with a healthcare provider [16]. The Swedish-developed Health Dialogue (HD) is a health conversation including an educative tool for visualisation of the risk score for different lifestyle habits and can be used for repeated measurements. This tool is a graphic colourful presentation (Health Curve) of each individual’s lifestyle habits, combining different risk levels (Fig. 1). The Health Curve is discussed using motivational interviewing by the health care staff, together with the patient, and a personalised action plan is decided upon.

A Swedish cohort study of 35-year-old patients being re-assessed with a HD after a mean follow-up time of 2.5 years, showed improvements in several lifestyle habits, such as less smoking, lower fat intake and higher physical activity [17]. Two following studies in the same cohort showed that the HD was a prognostic tool for assessing the risk of developing diabetes mellitus, cardiovascular disease, and cancer [18], as well as reduced mortality in long-term follow-up [19].

HD has not been aimed specifically at patients with mental illness, and the effect of a systematic approach with repeated HDs in patients with mental illness in primary care has not been previously studied.

The aim of this pilot study was to assess the feasibility of the process, time, and resource problems as well as the safety of the method for a larger-scale cohort study using HDs at repeated measurement visits in patients seeking primary care due to psychiatric problems (HEAlth Dialogues in patients with Mental Illness in Primary Care).

## Methods

### Participants and setting

Adult patients visiting a primary health care centre (PHCC) due to mental illness (depression, anxiety, sleep disorders or stress-related problems) were offered to participate in the study during the visit. Patient recruitment took place opportunistically (inclusion after a visit to a doctor or psychologist due to mental illness) and chronologically between October 2019 until November 2021.

Eligibility criteria in this pilot study were the same as in the planned main study and described in a previous publication [18]. All medical professionals at the PHCC had the possibility to recommend participation in the study and put patients on a waiting list created for this particular purpose. A healthcare professional (nurse or dietician) contacted the patients from the waiting list by telephone, mailed the written consent form to participate in the study and a lifestyle questionnaire. The patient was scheduled for measurements (including blood pressure), blood testing and a baseline visit for the HD. After the baseline visit, a following visit including a HD was planned for all recruited patients after 6–24 months for follow-up.

### Objectives

- To study the effects of the systematic approach with two repeated assessments using the HD in patients with mental illness on the cardiovascular risk in primary care (main study)
- To study the feasibility of the method (pilot study)

### Outcomes

#### Specific outcomes for the main study

Short follow-up (within 24 months):

1. Description of self-reported lifestyles (tobacco use, alcohol consumption, physical activity), metabolic markers (blood sugar, lipids, BMI, blood pressure, waist-hip ratio) and cardiovascular risk profile assessment at baseline and follow-up.
2. Proportion of patients with a change at follow-up in self-reported lifestyle habits, metabolic markers and cardiovascular risk profile (1–4) as described on the Health Curve.

Outcomes for long-term follow-up:

1. Diagnosis and date of onset of type 2 diabetes mellitus (ICD 10: E11) myocardial infarction (MI) (I21), ischaemic stroke (I63), death, cardiovascular death (ICD 10: I), venous thromboembolism (I82.0-I82.3, I82.8, I82.9 and I82.8W) and the composite outcome measure MACE (MI, stroke or cardiovascular death) (19) within 20 years from baseline.
2. Association between risk profile on the Health Curve and incidence of type 2 DM or cardiovascular event as described above.

#### Specific outcomes for the feasibility study

- Dropout rate (proportion of patients leaving the study before follow-up, the reasons for dropout) including difference in baseline measurements between patients remaining in the study until follow-up and patients that did not.
- Time to follow-up (proportion of patients followed between 6 and 24 months after baseline)
- Risk level improvement (proportion of patients with improved cardiovascular risk profile between baseline and follow-up)

### Intervention

A health assessment using a detailed questionnaire about dietary habits, physical activity, heredity, smoking, alcohol, stress, and mental illness was completed by the patient on a web-based formulary, before the scheduled HD. Prior to the HD, patients were also appointed for fasting blood sampling (plasma glucose, serum total cholesterol, high-density lipoprotein cholesterol [HDL-C] and low-density lipoprotein cholesterol [LDL-C]), measurement of blood pressure, waist and hip circumference, and body mass index (BMI). The assessment results in a visual colourful scale (Fig. 1, Health Curve) showing a risk assessment with gradually increasing risk levels from green (as no risk) to yellow, orange, and red (as the highest risk) [15, 20].

### Lifestyle factors

Each risk factor is graded into three or four risk levels, with level 1 indicating lowest risk and level 3 (i.e., dietary habits, psychosocial strain, mental stress, and tobacco) or 4 (i.e., physical activity, alcohol intake) indicating the highest risk for development of cardiovascular disease.

Use of tobacco was calculated as grams of tobacco smoked per day equivalent to cigarettes per day. Daily use of snuff generated risk point 2 for tobacco. Alcohol intake was calculated at 40% alcohol (cl) per week.

Dietary habits were studied using a validated special food frequency questionnaire [20]. Physical activity at leisure time was estimated using the mean energy expenditure and mean time activity per week, dividing the subjects into four activity groups [20]. The classification of psychosocial strain into risk points was based on questions about employment, family situation, economic status, social network, and presence of close confidants. Based on the patients’ answers, points were assigned: unemployment or threat of unemployment (two points); economic problems (one point); not being married or living alone (one point); and absence of a close confidant (one point) [20]. Four to 5 points gave the highest risk level of 3, the 2–3 points gave risk level 2, and < 2 points gave risk level 1. Information about self-rated mental stress during the last year was retrieved from a 50-mm visual scale, where 0 mm indicated no stress and 50 mm indicated maximal stress. Additional questions were asked about sleeping problems, general fatigue, depression, and anxiety, generating one stress point for every alternative [15].

During the HD, a healthcare worker (nurse or dietician) with special training using the method met the patient and provided individually tailored advice based on the patient’s risk profile on the health curve, such as advice for smoking cessation, physical activity on prescription, contact with a dietitian or a physiotherapist. Continued contact with a psychologist or physician was planned, if necessary, for psychiatric support or for the management of impaired blood glucose, high blood cholesterol, or blood pressure. Heritability for cardiovascular disease or diabetes, a non-modifiable risk factor, is also documented on the Health Curve, and discussed upon during the HD, as a part of the individual’s total risk assessment.

### Sample size

A power calculation was made for the pilot study: the HD visually grades different health parameters on a scale between 1 and 4; each parameter can have a different value. We assumed an expected positive change with at least one step on the scale of at least one parameter for each patient between the different measurements. We estimated that 90% of patients have at least one unhealthy lifestyle habit and expected a 10% change in the risk profile as above on the health curve, which gives a sample size of 86 patients with a strength of 80% and a significance level of 0.05. Due to a lack of studies in primary care focused on adherence to lifestyle counselling in mental illness patients, the outcome is chosen based on a Swedish study focused on lifestyle improvements in 35-year-old individuals participating in a screening programme with HD and follow-up after 5 years [13].

We estimated recruiting each week at least five eligible participants with mental illness, i.e., about 200 patients per year, and that half of them would potentially agree to participate in the study. We thus estimated to include about 100 patients in 1 year in the pilot study.

If the inclusion will continue as planned, we estimate to recruit a total of 400 patients by the end of 2023, from several PHCCs. Depending on the number of PHCCs that will agree to participate, the study will have enough power to detect an expected effect of the HD on patients’ lifestyle habits.

### Feasibility criteria

- Dropout: 50% was considered an acceptable dropout rate based on a previous study showing 42% dropout rate in patients without mental illness [17]. No difference was expected between patients remaining in the study and patients dropping out regarding age, sex, psychiatric diagnoses, lifestyle habits, or cardiovascular risk profiles at baseline.
- Time to follow-up: 90% of the follow-up visits performed within the planned time frame of 6–24 months.

Risk level improvement: a proportion of 10% of patients that improve the cardiovascular risk profile for the different lifestyle habits was considered acceptable. As all healthcare professionals at the PHCC were engaged in informing the patients about this study at the time of the contact due to a psychiatric problem, the recruitment rate was not possible to measure and was not assessed as a feasibility outcome of this pilot study.

### Statistical analysis

Baseline and follow-up data were analysed with descriptive statistics. Group comparisons between baseline variables in follow-up attendees (patients remaining in the study until follow-up) and non-attendees (patients only presenting at baseline) were analysed with Student’s *t* test for continuous variables and chi^2^ test for categorical variables. A *p* value < 0.05 was considered significant. SPSS version 27 (IBM Corporation®) was used for all statistical analyses.

## Results

### Participant flow

A total of 64 patients were included in the study between October 2019 until November 2021, with follow-up visits between September 2021 until December 2022. The study is conducted according to proposed guidelines for reporting results in pilot trials [21].

### Recruitment

**Fig. 2:**
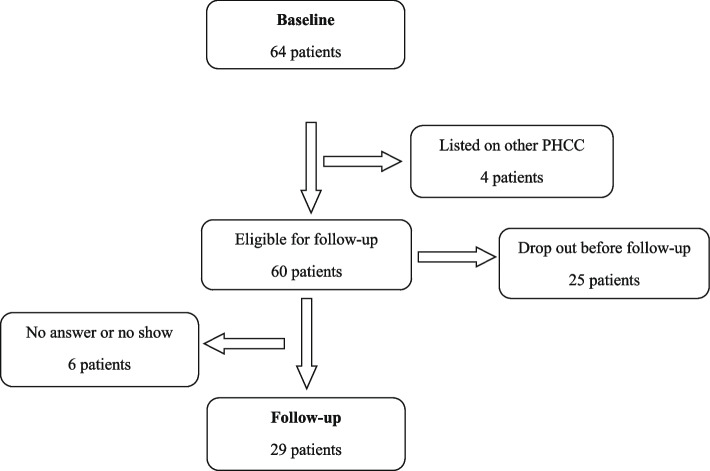
Flow description of patient recruitment and follow-up

Out of the included 64 patients at baseline, 29 patients (45%) attended the follow-up visit. The study flow is shown in Fig. 2.

### Baseline data

All patients had at least one psychiatric diagnosis, 27 patients (42%) had two diagnoses and 8 (12.5%) had even a third psychiatric diagnosis. Patients (47 women and 17 men) had a mean age of 52.7 years (women) and 49.7 years (men). 75.8% were overweight or obese, (mean BMI of 28.3 kg/m^2^ for the whole patient group), and 23.4% of all patients were very physically inactive. Baseline characteristics were presented in a study focusing on baseline data [22]. All participants had at least one elevated cardiovascular risk level on the Health Curve for the assessed lifestyles (measured on the scale between 1 and 3 or 4, where 1 is the lowest or no risk, and 4 is the highest risk), with no difference between men and women regarding the number of high or low-risk levels.

### Outcomes and estimations

A total of 29 patients (45%) attended the follow-up visit, leading to a dropout rate of 55%. Mean follow-up time was 15.5 months (median 13, range 6–29 months). A total of 76% percent of the patients (*N* = 22) attended the follow-up visit within 6–24 months, seven patients attended the second visit after 26 months (*N* = 3), 27 months (*N* = 3) or 29 months (*N* = 1) from baseline. There were no significant differences between follow-up attendee and non-attendee visits regarding baseline measurements (Table 1).

**Table 1:** Comparison of baseline measurements between attendees and non-attendees at follow-up

Variable at baseline	Follow-up attendees (*N* = 29)		Follow-up non-attendees (*N* = 35)		*p* value for difference
Age, mean	50.6		52.8		0.330^*^
Sex	Women, *N* (%) 18 (62)	Men, *N* (%) 11 (38)	Women, *N* (%) 29 (82)	Men, *N* (%) 6 (18)	0.061 ^**^
BMI	27.3		29.2		0.156^*^
Weight, mean (kg)	81.7		84.4		0.575^*^
Length, mean (cm)	172.5		169		0.112^*^
Waist-hip ratio, mean (cm)	0.92		0.93		0.888^*^
Waist circumference, mean (cm)	94.4		99.3		0.201^*^
Hip circumference (cm)	107.1		108.9		0.493^*^
Systolic blood pressure (mmHg)	128.2		124.6		0.293^*^
Diastolic blood pressure (mmHg)	80.7		78.6		0.327^*^
S-total cholesterol	5.9		4.8		0.358^*^
S-LDL-C^***^	3.9		3.2		0.434*
S-HDL-C^****^	1.7		1.5		0.401^*^
Fasting P-glucose	5.9		5.6		0.241^*^

^*^Student’s *t* test

^**^Chi^2^ test

^***^Low-density lipoprotein cholesterol

^****^High-density lipoprotein cholesterol

**Fig. 3:**
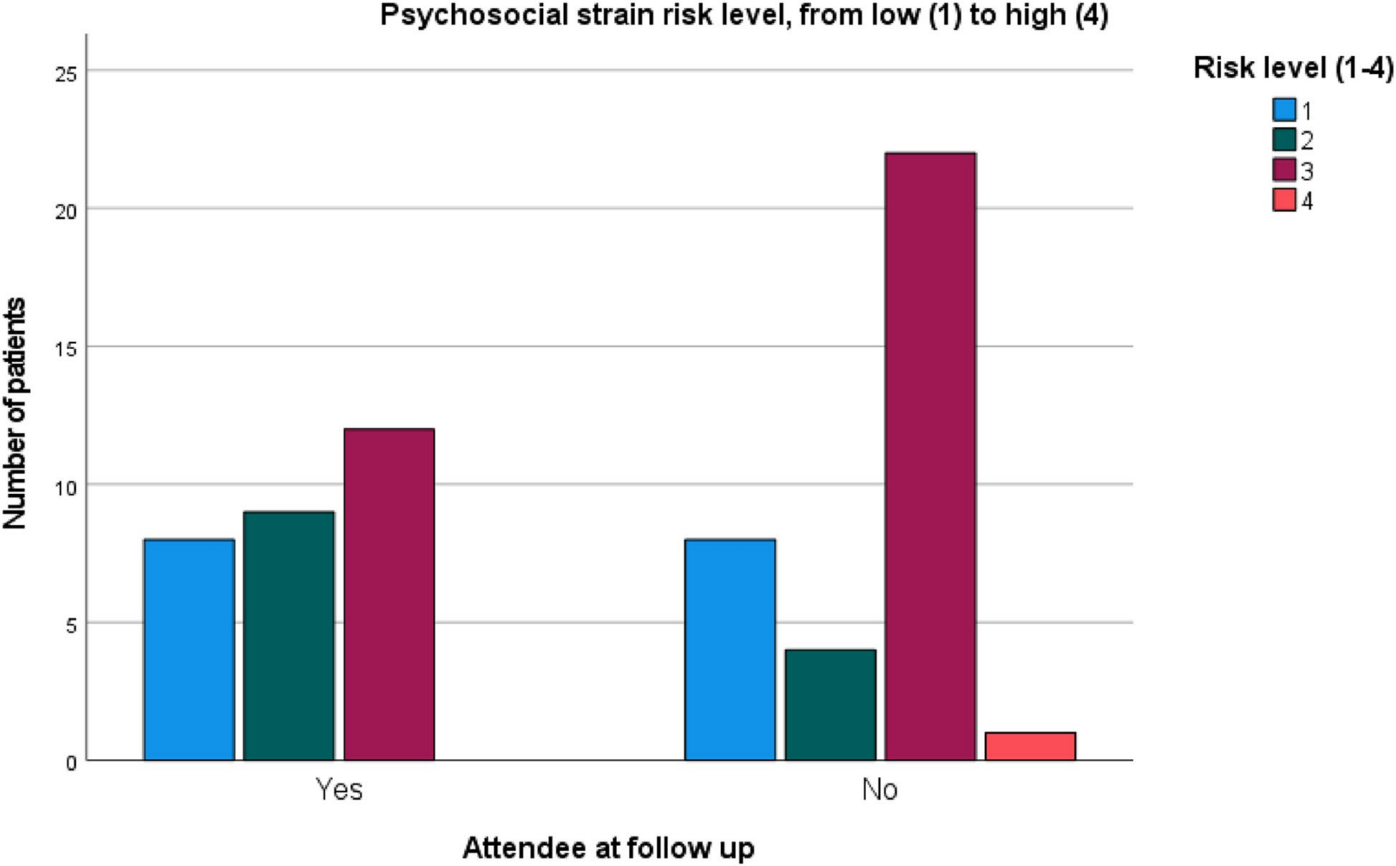
Bar chart with difference in psychosocial strain risk levels between follow-up attendees and non-attendees

The comparison of cardiovascular risk for the assessed lifestyle habits (Table 2) showed no statistically significant difference between the groups, except for one area: follow up non-attendees had a significantly higher risk for psychosocial strain reported at baseline (*p* = 0.030), compared with follow-up attendees (Fig. 3).

**Table 2:** Comparison of risk levels at baseline between follow-up attendees and non-attendees, chi^2^ test

	Follow-up attendees (*N* = 29) Risk level^*^				Follow-up non-attendees (*N* = 35) Risk level^*^				*p* value for difference
Risk factor	1	2	3	4	1	2	3	4	
Physical activity, *N*	8	10	5	6	6	12	8	9	0.751
Dietary habits	14	9	6	N/A	23	9	3	N/A	0.266
Alcohol intake	22	5	1	1	28	2	1	4	0.352
Tobacco use	24	3	2	N/A	27	3	5	N/A	0.635
Psychosocial strain	18	9	2	N/A	16	7	12	N/A	**0.030**
Mental stress	8	9	12	N/A	8	4	23	N/A	0.148

^*^Each risk factor is graded into three or four risk levels, with level 1 indicating lowest risk and level 3 or 4 indicating the highest risk for future development of cardiovascular disease

Risk level improvement was measured by calculating the percentage of individuals improving risk levels among follow-up attendees (Table 3). Regarding all lifestyle areas except tobacco use, more than 10% of the participants showed improvement in the risk level (feasibility outcome). In the physical activity and tobacco use lifestyle areas, the proportion of patients deteriorating on the Health Curve (with higher risk level at follow-up), was higher than the proportion of patients improving the risk level. In all other lifestyle areas, more patients improved than deteriorated their risk levels.

**Table 3:** Change in risk levels between baseline and follow-up visit

Risk factor	No change *N* (%)	Improved^*^ *N* (%)	Deteriorated *N* (%)
Physical activity (*N* = 29)	14 (48.3)	7 (24.1)	8 (27.6)
Dietary habits (*N* = 29)	19 (65.5)	6 (20.6)	4 (13.8)
Alcohol consumption^**^ (*N* = 28)	23 (79.3)	3 (10.3)	2 (6.9)
Tobacco use (*N* = 29)	24 (82.8)	2 (6.9)	3 (10.3)
Psychosocial strain (*N* = 29)	19 (65.6)	7 (24.1)	3 (10.3)
Mental stress (*N* = 29)	19 (65.6)	6 (20.6)	4 (13.7)

^*^Feasibility outcome

^**^Data for alcohol consumption was missing at follow-up for one patient

## Discussion

### Interpretation

The study had a high dropout rate (55%) between baseline and follow-up visits, with a lower percentage than expected (76%) of the follow-up visits performed within the pre-established time span of 6–24 months from baseline. An acceptable proportion of follow-up attendees adopted recommended changes in lifestyle habits. More than 10% of the participants improved risk levels in most lifestyle areas (except for tobacco use), and more participants improved rather than deteriorated their risk levels (except for tobacco use and physical activity). We interpret the high dropout rate as an effect of the slow patient flow, but also because of patients not prioritising the visits. Due to the outbreak of the COVID-19 pandemic, which reached a high spread in southern Sweden during the spring of 2020, the PHCC staff had to redirect their time and resources to other activities, such as vaccination programs, and visits of a less urgent nature were de-prioritised. This unexpected turn of events of global proportions affected the recruitment pace of patients in the study, thus leading to a delayed process as well as fewer patients than planned. We believe that applying the study design without dealing with a pandemic at the same time, should ensure a more rapid recruitment process.

Another reason for patients not to prioritise the visits might have been the difficulty for healthcare services to reach patients for preventive measures, which has been demonstrated in earlier studies [23, 24].

Our results are similar to findings in another study using HD in a healthy population of 35-year-old individuals with follow up after 5 years [15], in which the proportion of individuals with insufficient physical activity increased. However, the percentage of smokers decreased by 9.4%, indicating that a longer follow-up period might be needed to capture changes in tobacco use.

The follow-up time varied, also due to organisational barriers, the pandemic, or patients re-booking the follow- up visit several times. This may be seen as both a strength and a limitation, as the method should be adapted to the needs of the participants.

No difference was seen between follow-up attendees and non-attendees regarding baseline measurements and cardiovascular risk levels, except for the risk area of psychosocial strain with higher risk levels at baseline in the dropout group. These results imply that patients dropping out from the study before follow-up did not have higher cardiovascular risk levels, but might not be interested in further lifestyle support due to difficulties attending related to psychosocial distress or strained family situation.

### Generalisability

The method with repeated HDs has not been used before for patients with mental illness, therefore it is difficult to draw conclusions as to whether the method is better suited as a two-point screening of lifestyle habits in this population. A larger cohort study should perhaps answer the question. The study cohort is limited to a small sample size of patients recruited at one PHCC, therefore with low generalisability. The intervention program described in this paper was carried out at the same time as a larger study including screening of lifestyle habits with HDs in 40- and 50-years old adults in southern Sweden. As the intervention is implemented in southern Sweden as regular praxis, and several PHCCs have trained medical staff in performing the HDs, we estimate that broader inclusion of patients should be carried out without difficulties.

### Overall evidence of feasibility

The opportunistic method of inclusion with all staff engaged in informing the patients about the study, despite the difficulties to measure recruitment rate, might be more suitable for including patients with mental illness in primary care. We argue that general screening in the population with letters of invitation to a lifestyle assessment might not capture individuals with psychiatric problems, as previous studies showed that these patients have lower attendance to care [23, 24]. We believe that a study performed in regular praxis, without the challenges imposed by the COVID-19 pandemic on resource utilisation, would contribute to a more efficient and structured follow-up of study subjects. Patients remaining in the study showed a good risk level improvement rate, with more than 10% of the participants improving at least one lifestyle risk factor on the Health Curve at follow-up, except for tobacco use.

### Comparison with other studies

Positive lifestyle changes and a functioning social network can contribute to a faster recovery from mental illness [25]. Our results are comparable with the findings in the cohort study of 35-year-olds using the same method, which was performed in Sweden, with 326 included participants [17], where almost half of the participants did not show up at the follow-up visit. The previous study found a higher prevalence of smokers amongst follow-up non-attendees. In our study, there were more individuals with high-risk levels for tobacco use at baseline in the follow-up non-attendees group. A larger cohort study is needed, to verify the hypothesis that tobacco users among patients with mental illness in primary care might be difficult to motivate to tobacco use cessation using the HD.

The baseline analysis showed that more than half of the participants had the highest risk level in at least one of the studied lifestyle areas [22]. Despite the small sample size of this study, baseline data confirms the hypothesis that this group of patients needs closer attention regarding lifestyle areas that might affect the mental wellbeing as well as increase the risk to develop diabetes or cardiovascular disease. As HD has not previously been studied in a cohort of patients with mental illness, we estimate that the larger HEAD-MIP cohort study is motivated, to study the effect on unhealthy lifestyle habits in this group of patients. As we managed to recruit 64 patients out of the 86 needed according to the power calculation for the pilot study, we will include several primary health care centers, with medical staff trained in performing HDs, and prolong the recruitment period, to ensure the calculated sample size of 400 patients in the large cohort study.

Studies show that effective lifestyle interventions are indicated for people with mental illness [26]. A cohort study would add further evidence upon the effectiveness of a structured method with HDs in primary care on modifiable unhealthy lifestyle habits and cardiovascular risk factors in this patient group.

## Conclusion

The pilot study design for HDs in patients with mental illness showed a high dropout rate, no difference in risk levels between attendees and non-attendees at follow-up with a mean period of 15 months between the assessments, and acceptable risk level improvement, except for tobacco use. We suggest that the proposed methodology for a full cohort study within the general practice of patients with mental illness in primary care is both acceptable to practices and feasible.

## Data Availability

The dataset supporting the conclusions of the article is available from the corresponding author upon reasonable request.

## Abbreviations

HD: Health dialogue
HEAD-MIP: HEAlth Dialogue in patients with Mental Illness in Primary care
PHCC: Primary health care center
BMI: Body mass index
HDL-C: High-density lipoprotein cholesterol
LDL-C: Low-density lipoprotein cholesterol
MACE: Major adverse cardiovascular events
